# Cognition in older adults with healthy aging: analysis of the Mexican Health and Aging Study 2012–2015

**DOI:** 10.3389/fmed.2023.1207063

**Published:** 2023-07-06

**Authors:** Sara G. Yeverino-Castro, José D. Garza-Guerra, Gabriela E. Aguilar-Díaz, Célica R. González-Galván, Ricardo Salinas-Martínez, Rocío Morales-Delgado

**Affiliations:** ^1^Universitary Hospital “Dr. José E. González”, Geriatric Service, Universidad Autónoma de Nuevo León, Monterrey, Mexico; ^2^CHRISTUS Center of Excellence and Innovation, San Pedro Garza García, Mexico

**Keywords:** healthy aging, cognition, older adults, MHAS, CCCE

## Abstract

**Introduction:**

Maintaining older adults’ health and well-being can be achieved through the optimization of physical and mental health, while preserving independence, social participation, and quality of life. Cognitive change has been described as a normal process of aging and it involves domains such as processing speed, attention, memory, language, visuospatial abilities, and executive functioning, among others.

**Objective:**

To describe cognitive changes in older adults with healthy aging.

**Methods:**

This is a study that involved data from 14,893 and 14,154 individuals aged >60 years or older from the 2012 and 2015 waves, respectively, who participated in the Mexican Health and Aging Study (MHAS). Participants with healthy aging were identified and described in the MHAS-2012 wave and followed to 2015. Eight cognitive domains evaluated in the Cross-Cultural Cognitive Evaluation (CCCE,) as well as sociodemographic and health characteristics, were described. Criteria for healthy aging involved the following: CCCE ≥ −1.5 standard deviations above the mean on reference norms, independence on basic and instrumental activities of daily living, self-reported “life close to ideal,” and preserved functional and social performance.

**Results:**

From a total of *n* = 9,160 older adults from the MHAS-2012 wave, *n* = 1,080 (11.8%) had healthy aging. In the healthy aging group, the median age was 67 years (IQR: 63–73), 58.1% were female and the median for education was 6 (IQR: 3–8) years. The mean CCCE score was 57 (SD: 16.9) points. In the MHAS-2012 cross-sectional analysis, except for orientation, visuospatial abilities, and verbal fluency, all cognitive domain scores were lower with passing age. When comparing cognitive domain scores in the 225 older adults identified with healthy aging between the 2012 and 2015 MHAS waves, there were almost no observable differences.

**Conclusion:**

In the cross-sectional analysis, Mexican adults with healthy aging had lower scores in the verbal learning memory, visual scanning, numeracy, visual memory, and verbal recall domains’, as well as lower global cognitive scores in the higher age groups. There were no cognitive changes in the 3 year follow-up, except for a lower gradient of scores in the verbal recall memory domain. Longer prospective studies are needed to characterize greater cognitive changes.

## Introduction

Due to medical and technological advances along with better social and economic conditions, life expectancy has increased steadily around the world (1, 2). In 2015, it was estimated that the number of Mexican older adults will reach 150 million (3). Moreover, in 2020, life expectancy at birth in Mexico was estimated at 75.2 years (4, 5). However, healthy life expectancy was calculated at 65.4 years, evidencing a 10 year disparity between these two indicators (6, 7). The impact of the aging population, particularly in low- to middle-income countries, translates to an increase in multimorbidity, disability, and dependence, which represent a challenge for health systems (8, 9). Maintaining older adults’ health and well-being through the optimization of physical and mental health, while preserving independence, social participation, and quality of life, is essential (10).

Depending on the author, the concept of healthy aging has been approached in several ways. Authors have defined it as “active,” “successful,” “productive,” or “healthy” aging (11). Rowe and Kahn made an important contribution proposing a theoretical model of “successful aging,” at the individual level, that encompasses three different areas: disease and disability prevention, maintenance of high physical and cognitive function, and having a sustained commitment to social and productive activities (12–14). However, the World Health Organization (WHO) favors the term “healthy aging,” which focuses on functional abilities that result from the individuals´ interaction between their own intrinsic capacities and the environment (15).

Cognitive deterioration has been described as a normal process of aging, but also as part of other clinical conditions such as dementia (16). Normal cognitive changes have been well documented in several studies that describe domains such as processing speed, attention, memory, language, visuospatial abilities, and executive functioning, among others (16, 17). In Mexico, a pair of studies have described cognition as a part of the intrinsic capacity component of the WHO healthy aging definition. A study, based on data from the Mexican Health and Aging Study (MHAS)-2012 wave and a Mex-Cog 2016 study subsample, focused on describing the predictive value of the psychological and cognitive domains of the intrinsic capacity construct over successful memory aging (18). Similarly, Gutierrez-Robledo et al. evaluated intrinsic capacity in the MHAS-2015 wave and found that 88% of individuals had at least one of five domains affected (cognition, psychological, hearing, vision, vitality, and mobility) (19).

To gain a comprehensive understanding of cognitive function in non-demented community-dwelling older adults with healthy aging, it is necessary to describe a wide range of cognitive domains, given that their description in Mexican literature is warranted. The aim of our study is to describe cognition in older adults with healthy aging who participated in the Mexican Health and Aging Study (MHAS) 2012-wave and as secondary objectives, to determine healthy aging prevalence and to analyze cognitive changes between the 2012 and 2015 MHAS waves.

## Materials and methods

### Study participants and design

The MHAS is a national representative cohort study of Mexican adults aged 50 years or older (20). The baseline survey was conducted in 2001 with 5 follow-up waves in 2003, 2012, 2015, 2018, and 2021. We analyzed cross-sectional data from the MHAS-2012 wave and a 2015-wave subsample was used to fulfill one of the secondary objectives.

The MHAS description and ethical approval data are available at https://www.mhasweb.org/Home/StudyDescription.aspx and the aim and its methodological design is published elsewhere (21).

### Sample selection at baseline and follow-up

**Figure 1:**
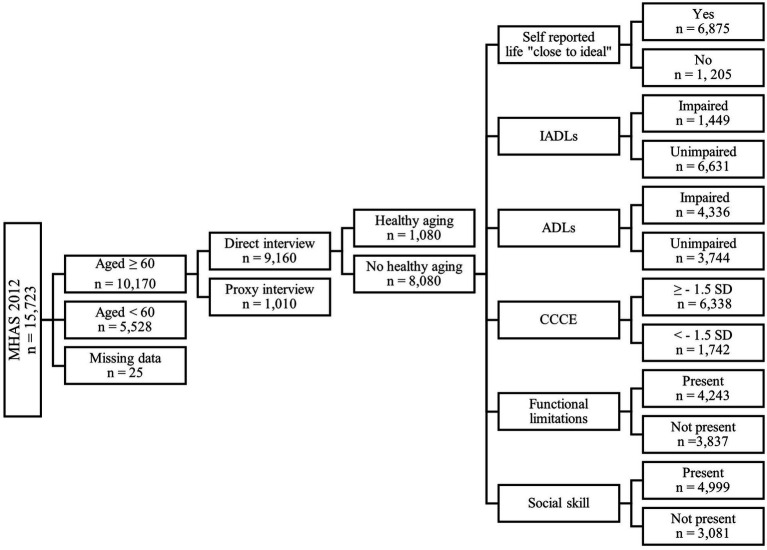
Flowchart of sample selection at baseline (MHAS-2012 wave). MHAS, Mexican Health and Aging Study; IADLs, instrumental activities of daily living; ADLs, activities of daily living; CCCE, Cross Cultural Cognitive Examination; SD, standard deviation.

Figure 1 shows the flowchart of the baseline sample selection. The 2012-MHAS wave included *n* = 15,723 participants that provided either direct or proxy interviews. From a total of *n* = 10,170 individuals aged 60 years or older, *n* = 9,160 with direct interviews were included. Individuals were further classified with (*n* = 1,080) or without healthy aging (*n* = 8,080). Individuals with healthy aging met all criteria; self-reported life “close to ideal,” unimpaired instrumental activities of daily living (IADLs) and activities of daily living (ADLs), a score ≥ −1.5 standard deviations (SD) in the Cross-Cultural Cognitive Examination (CCCE), absence of specific functional limitations, and presence of social skill.

**Figure 2:**
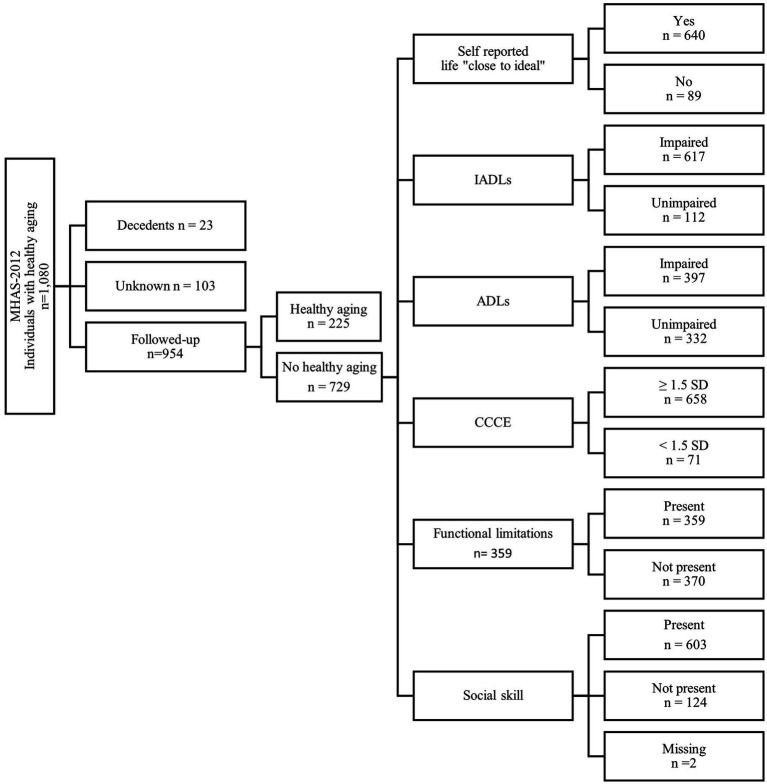
Flowchart of the followed-up sub-sample from the MHAS-2015 wave with and without healthy aging. MHAS, Mexican Health and Aging Study; IADLs, instrumental activities of daily living; ADLs, activities of daily living; CCCE, Cross-Cultural Cognitive Examination; SD, standard deviation.

To describe longitudinal cognitive changes, individuals with healthy aging (*n* = 1,080) were identified in 2012 and followed-up to 2015 (Figure 2). During follow-up, *n* = 23 individuals died (“decedents”) and *n* = 103 had unknown information. A total of *n* = 954 individuals comprised the followed-up sub-sample, which were further classified as with (*n* = 225) or without healthy aging (*n* = 729). Figure 2 shows the characteristics among those without healthy aging at follow-up, of which 90.3% had a score ≥1.5 SD in the CCCE, 87.8% had self-reported life “close to ideal,” 84.6% had unimpaired instrumental activities of daily living (IADLs), 54.5% had unimpaired activities of daily living (ADLs), and 49.2% remained with no specific functional limitations.

### Healthy aging

The WHO healthy aging definition comprises three fundamental principles: (a) functional capacity, which includes preserving the abilities that allow a person to fulfill basic needs, to learn and thrive, make decisions, establish relationships, contribute to society, and maintain mobility, (b) intrinsic capacity, which refers to a combination of a person’s physical and mental capacities, including the ability to walk, think, see, and remember, and (c) the environment factor, which involves people’s homes and their involvement in their communities (22).

In line with the mentioned criteria, individuals that fulfilled all of the following were classified with healthy aging: unimpaired IADLs (ability to prepare a meal, go shopping, manage money, or take medications), and ADLs (transferring or getting out of bed, dressing, toileting, grooming, or eating), scores in the CCCE ≥ −1.5 standard deviations (SD) above the mean based on previously published norms by age and education, absence of specific functional limitations (carrying objects, pushing or pulling, picking up a coin, or lifting arms), and presence of a social skill, defined by a positive response to the question: “*Does respondent communicate with relatives/friends via phone/internet?*” (23–26). In our study, self-reported life “close to ideal” based on the answer “agreed” to the question: “*Respondent believes his/her life is close to ideal?*,” was also considered. Supplementary Table S1 shows the healthy aging criteria used in this study in contrast to those proposed by the WHO (15).

### Cognition and cognitive domain evaluation

In an effort to identify non-demented individuals, as part of a healthy aging definition, individuals with CCCE scores ≥−1.5 SD above the mean based on reference norms by age and education were first identified (26). Individuals had unimpaired IADLs, which is essential when evaluating cognitive impairment (27).

Because other MHAS waves used a modified version of the CCCE, we used data from the 2012 and 2015 MHAS waves, in which a total CCCE score consists of a sum of maximum 99 points. As described by Mejía-Arango et al., the minimum and maximum scores for each cognitive domain are as follows: orientation 0–3, verbal learning memory 0–8, verbal recall memory 0–8, visual scanning 0–60, visuospatial abilities 0–6, visual memory 0–6, verbal fluency 1–4, and numeracy 1–4. Subsequently, each cognitive domain score was described.

### Covariables

Sociodemographic characteristics included age, sex, education, civil status, and religious service attendance. The latter was characterized by individuals who answered yes to the question: “*Respondent attends religious services?*.” Health characteristics included smoking history and current alcoholism, defined by a positive answer to the questions: “*Last 2 years: Respondent smoked cigarettes?*” and “*Respondent currently drinks alcohol?*,” respectively. Individual’s affirmative responses to the questions: “*Has a physician ever diagnosed you with [*i.e.*, hypertension, type 2 diabetes mellitus, cancer, heart attack, and rheumatoid arthritis]?*,” were also considered as comorbidities. Obesity was defined as a body mass index (BMI) ≥ 30 kg/m^2^ (28). We defined depressive symptoms according to a 9-item (yes/no) previously validated version of the Center for Epidemiological Studies-Depression (CES-D) included in the MHAS. A score ≥5 was considered as clinically significant depressive symptoms (29).

In this study, we included common geriatric syndromes such as the presence of falls, pain, stress and urge urinary incontinence, loss of appetite, hearing aid use, and visual impairment positive answers to the questions: “*Last 2 years: Has respondent fallen down?*,” “*Respondent suffers from pain?*,” “*Last 2 years: frequent incontinence while performing task(s)*?, “*Last 2 years: Frequent incontinence with urge to urinate*” “*Respondent uses hearing/auditory device*?,” respectively. Visual impairment refers to individuals who responded that they used glasses and had an “excellent-regular” vision with them.

### Statistical analysis

A Kolmorogov–Smirnoff test was conducted to determine the sample’s data distribution. In the cross-sectional analysis, median, interquartile ranges, and Mann–Whitney U tests were used to describe numerical variables and a Chi-square test was used for categorical variables. The total CCCE score was the only normally distributed variable and was described with means and *t*-student tests. Using information from the MHAS-2012 wave, box plots were constructed to show differences, by age group, between the median values of each cognitive domain and total CCCE scores. Domains with no visible cognitive change (orientation, visuospatial abilities, and verbal fluency) were not included. A prevalence rate was calculated in the MHAS-2012 wave. For the longitudinal analysis, information from individuals who fulfilled healthy aging criteria in both 2012 and 2015 (*n* = 225), were analyzed with the Wilcoxon signed-rank test and *t*-student paired test. Statistical significance was considered at a value of *p* ≤0.05 and analyses were performed using SPSS software for Windows^®^ (SPSS Inc., Chicago, IL version 23.0).

## Results

Sociodemographic, health characteristics, and presence of geriatric syndromes of the MHAS-2012 sample are shown on Table 1. From a total of 9,160 participants aged 60 years or older, 1,080 (11.8%) had healthy aging, median age was 68 years, most were women, and the median for education was 4 years. More than half of individuals were married and almost 80% of the sample said they attended a religious service. Thirty-one percent of participants had a history of smoking and 21.3% had current alcoholism. Almost half of the sample had hypertension, a quarter had diabetes mellitus, and 16.4% had rheumatoid arthritis. A history of previous heart attack and cancer diagnosis were present to a lesser extent. The most prevalent geriatric syndromes were the presence of falls, pain, and depressive symptoms, followed by visual impairment, both stress and urge urinary incontinence, loss of appetite, and hearing aid use.

**Table 1:** Sociodemographic, health characteristics, and presence of geriatric syndromes in the MHAS-2012 sample.

	Total *n* = 9,160	Healthy aging *n* = 1,080	With no healthy aging *n* = 8,080	*p* value^*^
Age median (IQR)	68 (64–75)	67 (63–73)	68 (64–75)	<0.001
Sex (female) (%)	54.5	58.1	54.0	0.013
Education median (IQR)	4 (1–6)	6 (3–8)	4 (1–6)	<0.001
Civil status (%)				0.190
Married	58.2	61.3	57.8	
Attends religious service (%)^**^	77.8	78.2	77.7	0.913
Smoking history (%)	31.0	32.0	30.9	0.614
Current alcoholism (%)	21.3	24.6	20.8	0.038
Obesity (%)	26.2	33.8	25.1	<0.001
Hypertension (%)	48.8	48.9	48.8	0.916
DM (%)	24.8	23.8	25.0	0.657
Cancer (%)	2.4	1.9	2.4	0.657
Heart attack (%)	4.3	4.0	4.3	0.875
RA (%)	16.4	13.1	16.8	0.013
Depressive symptoms (%)	33.4	25.6	34.5	<0.001
Geriatric syndromes				
Falls (%)	42.9	42.1	43.0	0.842
Pain (%)	39.4	37.2	39.7	0.245
Stress urinary incontinence (%)	15.7	15.6	15.7	0.784
Urge urinary incontinence (%)	16.2	16.2	16.2	0.960
Loss of appetite (%)^***^	6.2	3.6	6.5	<0.001
Hearing aid use (%)	1.5	1.5	1.4	0.931
Visual impairment^****^ (%)	10.8	6.7	11.4	<0.001

^*^Value of *p* from Mann–Whitney U test for numeric variables and Chi-square for categorical variables. IQR, interquartile range; DM, type 2 diabetes mellitus; RA, rheumatoid arthritis. ^**^Attends religious service analysis was performed with data from 1,142 participants who answered yes to the question: “*Respondent attends religious services*”. ^***^Loss of appetite refers to “frequently feeling with a loss of appetite.” ^****^Visual impairment refers to using glasses and having an excellent-regular vision with them.

When compared to the medians from the group without this characteristic, the healthy aging group was slightly younger and had a higher education (Table 1). Moreover, in the healthy aging group there was a statistically significant greater frequency of women, current alcoholism, and obesity, when compared to the group without it. The only comorbidities that were more prevalent in the group without healthy aging were depressive symptoms and rheumatoid arthritis. Loss of appetite and visual impairment were the less prevalent geriatric syndromes in the group with healthy aging. There were no significant differences between groups regarding other health characteristics.

Regarding the cognitive domain description between groups from the MHAS-2012 wave presented in Table 2, individuals with healthy aging had a higher global CCCE score and visibly greater individual cognitive domain scores in the verbal learning memory, visual scanning, numeracy, and verbal recall memory domains. When compared to the group without it.

**Table 2:** Cognitive domains description between the healthy and non-healthy aging groups in the MHAS-2012 sample.

Cognitive domain Median, (IQR)	Total *n* = 9,160	Healthy aging *n* = 1,080	With no healthy aging *n* = 8,080	*p* value^*^
Verbal learning memory	5 (4–5)	5 (4–6)	4 (4–5)	<0.001
Verbal fluency	2 (2–2)	2 (2–3)	2 (2–2)	<0.001
Visual scanning	21 (9–33)	27 (18–39)	20 (8–32)	<0.001
Orientation	3 (2–3)	3 (2–3)	3 (2–3)	<0.001
Numeracy	4 (3–4)	4 (3–4)	3 (2–4)	<0.001
Visuospatial abilities	6 (5–6)	6 (6–6)	6 (4–6)	<0.001
Visual memory	5 (2–6)	5 (4–6)	5 (2–6)	<0.001
Verbal recall memory	4 (2–5)	5 (3–5)	4 (2–5)	<0.001
Total CCCE, mean (SD)	46.2 (22.4)	57.0 (16.9)	44.8 (22.6)	<0.0.001

Value of *p* from Mann–Whitney U test for numeric non-parametric variables and *t*-student for parametric variables which refers to the total CCCE scores. IQR, interquartile range; CCCE, Cross Cultural Cognitive Examination; SD, standard deviation.

**Figure 3:**
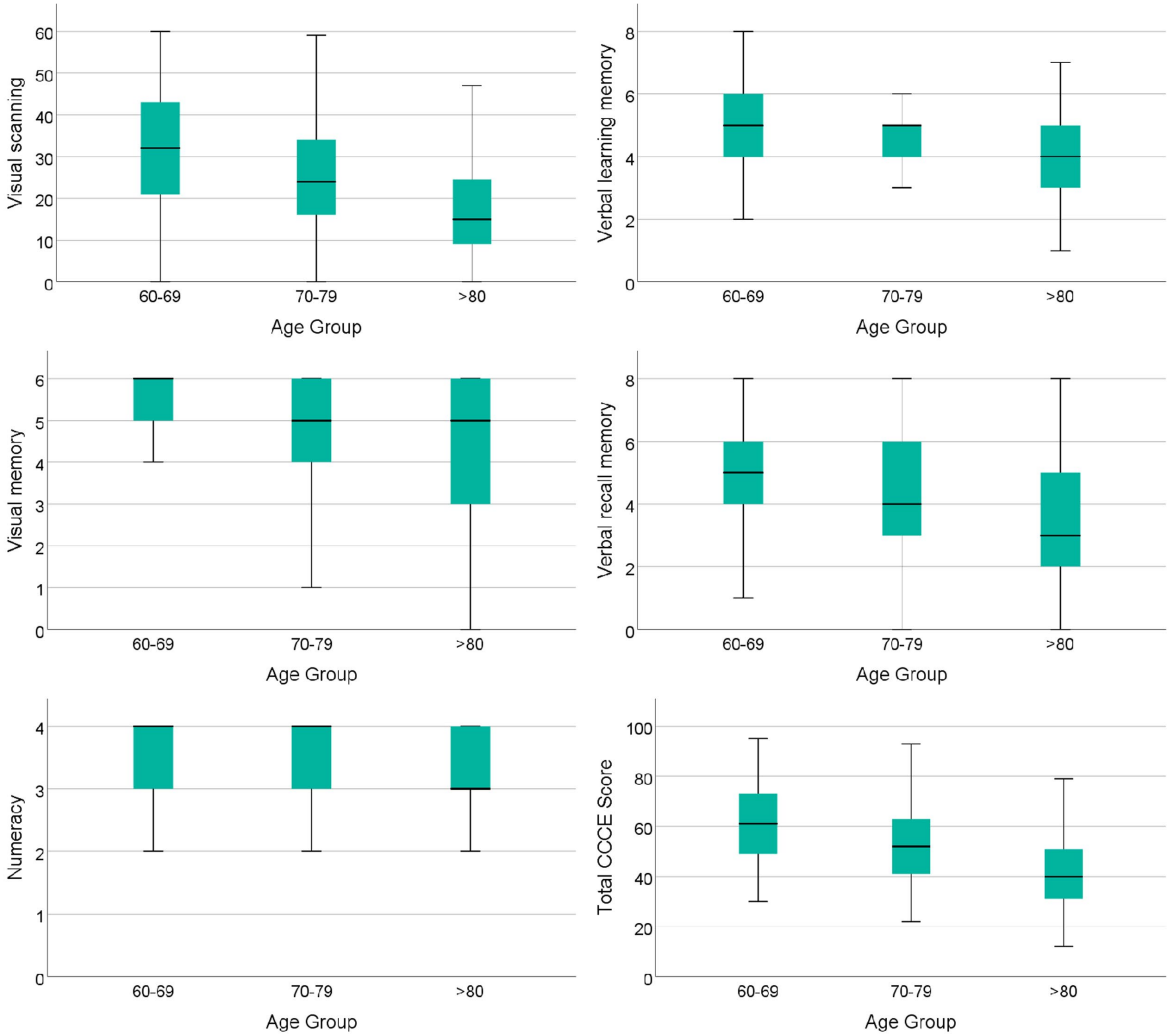
Box plots showing cognitive domain changes by age group in adults with healthy aging from the MHAS-2012 wave. *p* values from all comparisons between age groups in each cognitive domain and CCCE scores shown in the figure were <0.001. Individual cognitive domain comparisons were analyzed with a Kruskal–Wallis test and a *t*-student test was used for the total CCCE scores. CCCE, cross cultural cognitive examination.

The box plots showing cognitive domain changes by age group in the MHAS-2012 wave are presented in Figure 3. The median cross-sectional scores for all cognitive domains were visibly lower at older age, except for orientation, visuospatial abilities, and verbal fluency, thus, not included in the figure. The mean total CCCE score also had a significant decline. A detailed description of these variables is shown in Supplementary Table S2.

On Table 3, the cognitive domain changes observed between the 2012 and 2015 MHAS waves are shown. A total of 225 older adults survived and fulfilled healthy aging criteria in 2015. In this analysis, there were no observable differences among the assessed cognitive domain, except for the verbal recall memory domain interquartile range scores, which were lower in 2015 [5 (IQR: 3–6) points], when compared to 2012 [5 (IQR: 4–6) points] (*p* = 0.044), suggesting a gradient of changes in this domain in the 3 year follow-up. Supplementary Table S3 shows cognitive performance scores among the 954 individuals with healthy aging in 2012 that survived follow-up but did not fulfill healthy aging criteria. In this analysis the visual scanning, visual memory, and the verbal recall domains appeared to have lower scores in the 3 year follow-up.

**Table 3:** Cognitive changes between 2012 and 2015 MHAS waves.

Cognitive domain Median, (IQR)	Healthy aging 2012 *n* = 225	Healthy aging 2015 *n* = 225	*p* value^*^
Verbal learning memory	5 (4–6)	5 (4–6)	0.430
Verbal fluency	2 (2–3)	2 (2–3)	0.341
Visual scanning	30 (20–41)	31 (20–43.5)	0.079
Orientation	3 (2–3)	3 (2–3)	0.111
Numeracy	4 (3–4)	4 (3–4)	0.050
Visuospatial abilities	6 (6–6)	6 (6–6)	0.659
Visual memory	6 (5–6)	6 (5–6)	0.294
Verbal recall memory	5 (4–6)	5 (3–6)	0.044
CCCE, mean (SD)	60 (48–71)	60 (48–75)	0.391

^*^p-value from Wilcoxon signed-rank test between healthy aging participants from the 2012 and 2015 MHAS waves. ^**^p-value from paired *t*-student test. CCCE, Cross Cultural Cognitive Examination; SD, standard deviation.

## Discussion

In the cross-sectional analysis by age group from the MHAS 2012-wave, we found that cognitive domains such as visual scanning, verbal learning memory, visual memory, verbal recall memory, numeracy, and total CCCE scores were lower at older ages. These changes were not evident in the 3 year follow-up analysis among individuals who fulfilled healthy aging criteria. An 11.8% (CI: 11.1–12-4) prevalence of healthy aging in adults aged 60 years or older was found in the 2012 MHAS-wave.

Different concepts have been proposed when defining healthy aging. Rowe and Khan model of successful aging is one of the most used by several authors (12–14). For instance, a study involving 14 European countries established a “successful” aging prevalence of 8.5% (30). In China, Yin et al. found a prevalence of 15.8% and a 12.6% frequency was reported in western Mexico in 2012 (31, 32). The latter prevalence is like that found in our study (11.8%) but lower than that reported in China. It is important to note that we used the WHO healthy aging definition which does not consider the strict absence of comorbidities (22). Additionally, our definition included a “life close to ideal” self-perception criterion, as it has been considered beneficial to functional health and described as a predictor of future morbidity and mortality in numerous empirical studies but, nevertheless, is not included as essential in the two concepts previously mentioned (33, 34). Moreover, cognition in our study was thoroughly evaluated with a different instrument, which has proven useful in cross-cultural epidemiological research, from that included in other studies (35). The strictness and number of criteria considered to establish healthy or successful aging should be considered when comparing results and could account for differences or similarities in the prevalence rates reported.

Unlike developed countries, Mexico’s population is still undergoing a demographic transition in which people over 65 years of age are expected to greatly increase in proportion by 2050 (36). Regarding the age characteristics of our study’s sample, the healthy aging group had a median age of 67 (IQR: 63–73) years, lower than the means previously reported in Europe (74.0 ± 3.8) and the United States of America (72.41 ± 8.47). Our results are only comparable to the findings by Arias-Merino et al. in Mexico, in which a greater frequency of successful aging (18.9%) was found in the 60–69 age group, when compared to older groups (32). Like what was reported by Schietzel et al. in European countries, the female sex was the most prevalent in the healthy aging group, while being married was the most common civil status, similar to that reported in other studies, probably because it has been suggested that marriage provides social benefits and has been previously associated with health and survival in the older age (31, 37–40). The group with healthy aging also had a higher level of education compared to the group without healthy aging, as reported by Schietzel et al (37). However, consistent with what has been reported in developing countries, the mean for years of education was higher in the latter study (13.4 ± 3.5), when compared to the median value reported in our study [6 (IQR: 3–8) years, (41)].

Our findings were like the results described by Cañedo et al. in Brazil, in which healthy aging individuals had a BMI in the overweight range (34%) and 21% were cataloged with obesity (38). Bowling et al., found a possible null relationship between overweight and mortality in older adults and in another study, it was found that neither overweight nor obesity were associated with mortality in univariate and multivariate models (42, 43). Authors in the latter study concluded that being underweight, unlike being overweight or obese, increased the risk of premature death in older people (44). As expected from a country with a reported high prevalence of overweight (49.4%) and obesity (28.7%) and as a result of including comorbidities in the healthy aging definition, in our study, obesity was higher in the healthy aging group (45).

We did not find differences between individuals with or without healthy aging with respect to the presence of chronic diseases such as hypertension, diabetes mellitus, or heart attack. The presence of comorbidities has been identified as one of the most demanding criteria to define successful aging (12–14, 46). However, this would hardly apply to our population since chronic diseases are prevalent in Mexico due to socioeconomic conditions and the overall level of sub-development (47). Moreover, it has been described that successful aging can coexist with chronic diseases and functional limitations if sufficient compensatory mechanisms exist (48). Additionally, it has been observed that the preservation of functionality associated with a good self-perception of health is possible in the presence of comorbidities (38).

Regarding geriatric syndromes, individuals with healthy aging in our study had a lower presence of depression and loss of appetite. A previous study by Cañedo, et al. found that depression is associated with fewer social engagement and less physical activity, which causes greater functional deterioration (38, 49). In addition, as previously described, individuals with visual impairment have a higher risk of functional impairment, which could explain our results (50).

In the cross-sectional analysis, by age group, we found lower cognitive domain scores in higher age groups. The CCCE used in the MHAS evaluates verbal memory (learning and recall) through an eight-word list (26). We found that both domains (verbal learning memory and verbal recall memory) scores seemed to be lower at older age. Similarly, Young Hoogendam et al., in an analysis of the Rotterdam Study, found that compared to other domains, the smallest, but present, effects of age over performance were found in the immediate and delayed recall word tests (16). Furthermore, the CCCE assesses working memory with the use of the numeracy domain (counting backwards from 20 to 0 in a maximum time of 60 s) (51). We also observed lower scores in this task with passing age. Described as produced by a change in frontal-striatal circuits, executive function has been found reduced in older adults without cognitive impairment along with working memory (17, 52).

Attention, evaluated in the CCCE through a visual scanning task (detecting stimuli among other similar stimuli), was also found reduced with advancing age (51). Age has been found to have a more significant effect on complex attention tasks such as selective attention, which involves the capacity to focus on specific information while ignoring irrelevant stimuli (17). Similarly, the most noticeable difficulties described in older adults above their ninth decade of life, were cognitive slowing and diminished attention skills (52). On a separate note, visual memory was measured in the MHAS by requesting individuals to remember figures they had previously copied (51) and it was also found reduced with older age. Verbal and visual working memory have both been found in like manner affected by normal aging (53). Total CCCE scores in older adults with healthy aging were also lower at higher age groups in the cross-sectional analysis. The Rotterdam study reported rapid cognitive decline in global Mini-Mental State Examination (MMSE) scores after the age of 70 (16).

In our study, the orientation (knowledge about the day, month, and year), visuospatial abilities, and verbal fluency (animal naming for 1 min) (26) domains had no visible change with advancing age. Unlike the Rotterdam Study in which a decline in the verbal fluency and visuospatial abilities (copy two figures), mostly the latter, were found affected by age (16). The latter was also like the findings of Harada et al., who reported that visual construction skills decline with age, in contrast to familiar object recognition and spatial perception which remain unchanged with age (17). Lastly, temporal orientation is considered a reflection of semantic and episodic information (54). It has been found that while semantic memory remains relatively stable with advancing change, a reduced episodic memory has been associated with aging (55, 56).

A decline among cognitive domains evaluated in adults aged 60 years or older that participated in the MHAS was not evident in the 3 year follow-up analysis, except for a slight IQR lower score in the verbal recall memory domain. Other longitudinal studies, with greater follow-ups, have reported a similar decline on immediate and delayed recall tests (16, 57), highlighting the need for longer assessments.

This study has several strengths. First, to the best of our knowledge, it is the first study in Mexico that evaluates healthy aging using the WHO definition, which does consider the presence of comorbidities. Second, a cross-sectional and longitudinal analysis was performed describing changes over time in cognitive domains of individuals with healthy aging. Third, in our study we used the CCCE for the evaluation of cognitive domains, which evaluates multiple cognitive areas, unlike other more commonly used scales (32, 58). Fourth, our study is based on a large representative sample of the Mexican population.

Our study is not without weaknesses. First, we performed a 3 year follow up which was not enough to detect significant changes among the cognitive domains evaluated. A longer follow-up period is warranted. Second, perhaps because our criteria for healthy aging were too strict, our sample size was smaller than it would have been if functional capacity had not been evaluated as it was. Third, we must consider that other studies measured cognitive domains with broader neuropsychological tests, which could account for the difference in results (59).

## Conclusion

Cross-sectionally, this study shows cognitive domain changes, concerning lower scores in the higher age groups in the visual scanning, verbal learning memory, visual memory, verbal recall memory, and numeracy domains, of Mexican older adults with healthy aging that participated in the MHAS-2012 wave. However, changes were not observed in the 3 year longitudinal analysis, hence a longer follow-up is warranted to better describe changes through time.

## Data availability statement

The raw data supporting the conclusions of this article will be made available by the authors, without undue reservation.

## Author contributions

SY-C, RM-D, and JG-G designed the study. SY-C, RM-D, CG-G, and JG-G searched the literature. SY-C, GA-D, JG-G, and RM-D collected and analyzed the data. SY-C and GA-D interpreted the data. SY-C and RM-D wrote the manuscript draft. All authors contributed to the article and approved the submitted version.

## Funding

The dissemination and publication of the results obtained from this work were supported by CHRISTUS Center of Excellence and Innovations, San Pedro Garza García, Nuevo León, México. and the Geriatric Service of the Universitary Hospital “Dr. José Eleuterio González” Universidad Autónoma de Nuevo León, Monterrey, Nuevo León, Mexico.

## Conflict of interest

The authors declare that the research was conducted in the absence of any commercial or financial relationships that could be construed as a potential conflict of interest.

## Publisher’s note

All claims expressed in this article are solely those of the authors and do not necessarily represent those of their affiliated organizations, or those of the publisher, the editors and the reviewers. Any product that may be evaluated in this article, or claim that may be made by its manufacturer, is not guaranteed or endorsed by the publisher.
