# Supplementary material for: Cognition in older adults with healthy aging: analysis of the Mexican Health and Aging Study 2012–2015
Source: Front Med (Lausanne). 2023 Jul 6;10:1207063. doi: 10.3389/fmed.2023.1207063 (PMC10361761; doi:10.3389/fmed.2023.1207063)
Supplement: Supplementary file 1 [file Table_1.docx]

**Supplementary Table 1.** Criteria for Healthy Aging.

| Variable | Evaluation | WHO principle* |
| --- | --- | --- |
| Unimpaired cognition | A score in the CCCE* ≥ - 1.5 SD. | **Intrinsic capacity**  The combination of physical and mental capacities. |
| Unimpaired IADLs | A negative answer to all the following questions: *Does anyone help you prepare a hot meal? Does anyone help you shop for groceries? Does anyone one help you manage your money? Does anyone help you take medications?* | **Functional ability**  Characteristics that make it possible for people to be and do what they want). Functional ability also involves an interaction between the individual’s intrinsic capacities and relevant environmental factors. |
| Unimpaired ADLs | A negative answer to all the following questions: *Does someone help you: get into or out of bed, use toilet, get on off, to bathe or shower, eat your food?* *Because of health problem, difficulty dressing self?* | **Functional ability**  Preserving an individuals’ autonomy involves “being independent”. |
| Absence of specific functional limitations | A negative answer to all the following questions: *Because of health problem, difficulty carrying objects, pushing, or pulling, difficulty picking up a coin, lifting arms?* | **Functional ability** In order to maintain an individual’s role of identity and autonomy, among other attributes, “the ability to move around is crucial”. |
| Self-reported “life close to ideal” | *A positive answer to the question: Respondent believes his/her life is close to ideal?* | **Well-being** This feature includes characteristics such as “happiness, satisfaction, and fulfillment”. |
| Social skill | *A positive answer to the question: Does respondent communicate with relatives/friends via phone/internet)”* | **Environment**  Contextual components of the individual’s “extrinsic world including – home, communities, and society”. |

CCCE: Cross Cultural Cognitive Examination**,** IADLs: instrumental activities of daily living, ADLs: activities of daily living, SD: Standard deviation. *Based on the WHO healthy ageing definition published in 2015.

**Supplementary Table 2**. Cognitive domain description by age group in individuals with health aging from the MHAS-2012 wave.

| Variables | Healthy  Aging 2012  Total | Age groups | | | | P-value |
| --- | --- | --- | --- | --- | --- | --- |
| Median (IQR) | n= 1.080 | **60-69**  n= 660 | **70-79**  n= 337 | | **>80**  n= 83 |  |
| Verbal learning memory | 5 (4-6) | 5 (4-6) | 5 (4-5) | 4 (3-5) | | <0.001 |
| Verbal fluency | 2 (2-2) | 2 (2-3) | 2 (2-2) | 2 (2-2) | | <0.001 |
| Visual scanning | 27 (18-39) | 32 (21-43) | 24 (16-34.5) | 15 (9-25) | | <0.001 |
| Orientation | 3 (2-3) | 3 (2-3) | 3 (2-3) | 3 (2-3) | | <0.001 |
| Numeracy | 4 (3-4) | 4 (3-4) | 4 (3-4) | 3 (3-4) | | <0.001 |
| Visuospatial abilities | 6 (6-6) | 6 (6-6) | 6 (6-6) | 6 (5-6) | | <0.001 |
| Visual memory | 5.5 (4-6) | 6 (5-6) | 5 (4-6) | 5 (3-6) | | <0.001 |
| Verbal recall memory | 5 (3-6) | 5 (4-6) | 4 (3-6) | 3 (2-5) | | <0.001 |
| CCCE mean (SD) | 56 (44-70) | 61 (49-73) | 52 (41-63) | 40 (31-51) | | <0.001 |

P-value from Kruskal-Wallis test and CCCE: Cross Cultural Cognitive Examination, SD: standard deviation. All values ≥ to the median*.

**Supplementary Table 3**. Cognitive changes between 2012 and 2015 MHAS waves of the 954 individuals who had healthy aging in 2012.

| Cognitive domain  Median, (IQR) | Healthy  aging  2012  n= 954 | Follow-up 2015     n= 954 | P value* |
| --- | --- | --- | --- |
| Verbal learning memory | 5 (4-6) | 5 (4-6) | 0.004 |
| Verbal fluency | 2 (2-3) | 2 (2-3) | 0.646 |
| Visual scanning | 27 (18-39) | 26 (16.7-39) | 0.001 |
| Orientation | 3 (2-3) | 3 (2-3) | <0.001 |
| Numeracy | 4 (3-4) | 4 (3-4) | <0.001 |
| Visuospatial abilities | 6 (6-6) | 6 (6-6) | 0.008 |
| Visual memory | 6 (4-6) | 5 (4-6) | <0.001 |
| Verbal recall memory | 5 (3-6) | 4 (3-6) | <0.001 |
| CCCE mean (SD) | 57.2 (16.8) | 54.6 (19.8) | <0.001 |

*P-value from Wilcoxon signed-rank test between healthy aging participants from the 2012 and 2015 MHAS waves. **p-value from paired T-student test. CCCE: Cross Cultural Cognitive Examination, SD: Standard deviation.
